# Cell Growth on Different Types of Ultrananocrystalline Diamond Thin Films

**DOI:** 10.3390/jfb3030588

**Published:** 2012-08-16

**Authors:** Bing Shi, Qiaoling Jin, Liaohai Chen, Amina S. Woods, Albert J. Schultz, Orlando Auciello

**Affiliations:** 1 Materials Science Division, Argonne National Laboratory, 9700 S. Cass Ave, Argonne, IL 60439, USA; Email: auciello@anl.gov; 2 Advanced Photon Sources, Argonne National Laboratory, 9700 S. Cass Ave, Argonne, IL 60439, USA; Email: qjin@anl.gov; 3 Bioscience Division, Argonne National Laboratory, 9700 S. Cass Ave, Argonne, IL 60439, USA; Email: l.chen@usu.edu; 4 Department of Chemistry & Biochemistry, Utah State University, Logan, UT 84322-03004, USA; 5 Structural Biology Unit, National Institute on Drug Abuse, NIH, Baltimore, MD 21224, USA; Email: awoods@mail.nih.gov; 6 Ionwerks Inc., Houston, TX 77005, USA; Email: al@ionwerks.com

**Keywords:** ultrananocrystalline diamond (UNCD), cell culture, biocompatibility, cell adhesion and proliferation

## Abstract

Unique functional materials provide a platform as scaffolds for cell/tissue regeneration. Investigation of cell-materials’ chemical and biological interactions will enable the application of more functional materials in the area of bioengineering, which provides a pathway to the novel treatment for patients who suffer from tissue/organ damage and face the limitation of donation sources. Many studies have been made into tissue/organ regeneration. Development of new substrate materials as platforms for cell/tissue regeneration is a key research area. Studies discussed in this paper focus on the investigation of novel ultrananocrystalline diamond (UNCD) films as substrate/scaffold materials for developmental biology. Specially designed quartz dishes have been coated with different types of UNCD films and cells were subsequently seeded on those films. Results showed the cells’ growth on UNCD-coated culture dishes are similar to cell culture dishes with little retardation, indicating that UNCD films have no or little inhibition on cell proliferation and are potentially appealing as substrate/scaffold materials. The mechanisms of cell adhesion on UNCD surfaces are proposed based on the experimental results. The comparisons of cell cultures on diamond-powder-seeded culture dishes and on UNCD-coated dishes with matrix-assisted laser desorption/ionization—time-of-flight mass spectroscopy (MALDI-TOF MS) and X-ray photoelectron spectroscopy (XPS) analyses provided valuable data to support the mechanisms proposed to explain the adhesion and proliferation of cells on the surface of the UNCD platform.

## 1. Introduction

Worldwide research is being performed to achieve regeneration of tissues and organs to repair diseased or damaged tissues and organs in the human body [1,2], which are in limited supply. This bioengineering approach provides treatment by growing regenerative tissue/organs on artificial substrates *in vitro* and implanting them back into the damaged areas of the body. Investigation of biomaterials as artificial platforms for cell growth and differentiation is one of the most important areas in regenerative medicine. Results from previous research suggest that it is possible to engineer growing tissue by presenting appropriate growth stimuli from the cell transplantation scaffolds and it is critical to promote the multiplication of transplanted cells if one is to engineer a growing tissue *in vivo*. A required growth stimulus for most mammalian cell types is needed for an appropriate adhesive substrate. Large-scale cell culture systems will be important to grow sufficient cells *in vitro* [3,4]. Therefore, materials used as adhesion substrates are a major area of study. Biomaterials used in tissue-engineering need to meet the nutritional and biological needs of the cells. They must have good mechanical characteristics and good geometries while the devices are fabricated so as to maintain the structure during new tissue formation. 

Most of the past research has focused on investigating several types of polymer biomaterials as scaffolds for bioengineering studies, namely: (a) natural materials such as collagen or alginate, which are biocompatible macromolecules that facilitate cell attachment or maintenance of differentiated function; and (b) synthetic materials such as lacticglycolic acid or polyacrylonitrile-polyvinyl chloride, that were used as templates for cells to form permanent new tissues [5]. However, the materials mentioned above may not interact with cells in a positive manner. The combination of the natural and synthetic materials uses critical amino acid sequences from natural polymers that are grafted onto synthetic polymers [6]. The microstructure and the surface chemistry of the materials are critical and need to be studied in detail, including investigation of issues such as producing a certain surface topography or porous microstructure that is similar to the extra-cellular matrix to get an engineered tissue response with the substrates [7]. Many materials [8] other than polymers have also been investigated such as carbon materials, including diamond-like carbon (DLC) [9,10,11], microcrystalline diamond, and nanocrystalline diamond [12,13,14]. 

Alternative to the materials mentioned above, in this paper we report our studies of cell growth on different types of UNCD surfaces as well as on diamond-powder-seeded surfaces. The difference between UNCD and other nanocrystalline diamond films is that UNCD films are synthesized using an Ar-rich/CH_4 _gas mixture [15,16] while other nanocrystalline diamond films are synthesized in a hydrogen-rich/CH_4_ gas mixture. UNCD films have unique microstructures that have a grain size of 3–5 nm for undoped UNCD and larger grain sizes for H-doped and N-doped UNCD, depending on the doping percentage. Because of its excellent mechanical properties such as hardness, low friction, and high anti-wear rate, as well as chemical inertness, UNCD has potential application as a unique biomaterial for implantable biomedical devices. In this study, different types of UNCD thin films were synthesized. Cell culture cytotoxicity of these different types of UNCDs was investigated. Results showed that all types of UNCD thin films could support cell growth. The microstructures of different UNCD films were analyzed, cell growth on different types of UNCDs were observed, and the mechanisms of cell adhesion are proposed. Analysis using matrix-assisted laser desorption/ionization—time-of-flight mass spectroscopy (MALDI-TOF MS) and X-ray photoelectron spectroscopy (XPS) provided valuable information to support the mechanisms proposed to explain the cell-UNCD interaction. 

## 2. Experiments

### 2.1. Synthesis of UNCD Films

The substrates used to grow UNCD films were first seeded with ultrananocrystalline diamond powder dissolved in methanol in an ultrasonic bath. Different UNCD films were synthesized in a microwave plasma chemical vapor deposition (MPCVD) system on quartz plates that were heated to 800 °C. Undoped UNCD films were grown using an Ar (99%)/CH_4_ (1%) gas mixture; H-doped UNCD films were grown using an Ar (97%)/CH_4_ (1%)/H_2_ (2%) gas mixture, and N-doped UNCD thin films were grown using an Ar/CH_4_/N_2_ gas mixture with N_2_ (10% and 20%). The UNCD films were exposed to H-plasmas to get H-terminated films: H-plasma-treated undoped UNCD and H-plasma-treated N-doped UNCD thin films.

### 2.2. Cell Culture

These UNCD-film-coated dished were sterilized with 70% ethanol followed by UV irradiation. Mouse embryonic fibroblasts (MEFs, Chemicon International) were first grown on normal culture flasks. When 80% confluency was reached, the cells were dissociated by Trypsin/EDTA and passaged onto quartz dishes (control), diamond-seeded dishes, and different UNCD-coated dishes at the same starting density of 8.0 × 10^3^ cells/cm^2^. Cell culture vessels were incubated at 37 °C in a humidified atmosphere containing 5% CO_2_. Cell growth was monitored daily for four days using optical microscopy. The experiments were repeated three times. 

## 3. Results

### 3.1. Seeding

**Figure 1:**
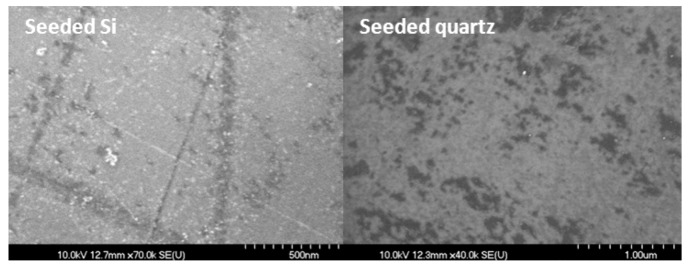
Scanning electronic microscopy (SEM) images of nanodiamond seeded on Si (**left**) and quartz (**right**) by mechanical seeding plus ultrasonic seeding procedures.

The seeding procedure resulted in nanodiamond seeds being embedded on the substrate surfaces which induced the nucleation and subsequent growth of the UNCD films. Polishing seeding plus an ultrasonic seeding procedure introduced a higher density of the seeds on the surface compared with using one seeding method alone. Scanning electronic microscopy (SEM) images (Figure 1) show a layer of nanodiamond powder on the surface of the Si substrate after seeding. Scratches are observed on the surface of the Si from the seeding procedure. 

### 3.2. Synthesis of UNCD Films

**Figure 2:**
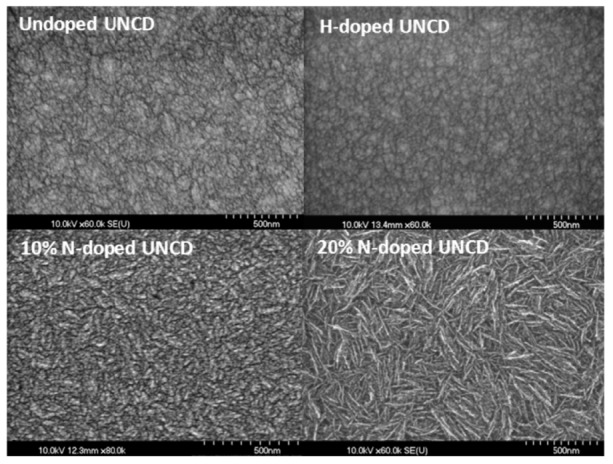
SEM images of different ultrananocrystalline diamond (UNCD) films.

Undoped UNCD, H-doped UNCD (1%), N-doped UNCD (10%), and N-doped UNCD (20%) thin films were synthesized. Scanning electronic microscopy (SEM) analysis images of these films are shown in Figure 2. 

Compared with undoped UNCD thin films, 1% H-doped UNCD thin films are less conductive and showed more charging during the SEM analysis. This is because the hydrogen atoms were inserted into the grain boundaries, suppressing the formation of sp^2^-bonded carbon; and the hydrogen satisfied the dangling bonds and formed H-terminated surface. N-doped UNCD thin films are semi-metallically conductive, which has been widely studied [15], and also could be seen during the SEM analysis procedure. Unlike H-doped UNCD films, which still have a similar surface morphology to undoped UNCD, N-doped UNCD showed a more needle-like pattern on the surface. The larger the N-doping percentage in the gas mixture, the longer the needles. However, AFM and TEM studies showed that N-doped UNCD had nanograins similar to undoped UNCD, which will be reported later. 

The UNCD films were exposed to H-plasma to achieve H-terminated UNCD films and H-terminated N-doped UNCD films. H-plasma treatment on UNCD resulted in H atoms bonding on the surface and diamond surface H-terminated [15,17].

### 3.3. Cell Culture

Cell growth on UNCD thin-film-coated and uncoated quartz dishes was observed daily. The three repeated samples showed similar results. Except for the quartz dishes, which showed cells that were almost totally detached from the dishes, all other substrate surfaces coated with UNCD films showed that cells attached to the surface and proliferated at different levels.

**Figure 3:**
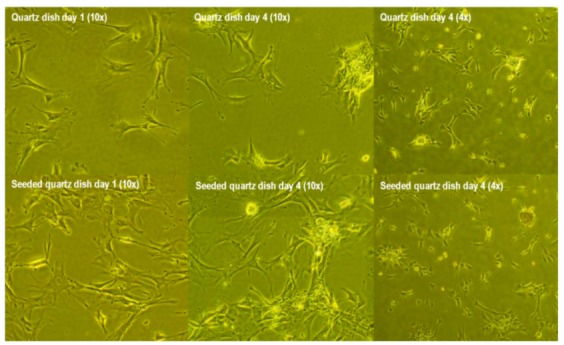
Mouse embryonic fibroblasts (MEF) growth on quartz dished and diamond-powder-seeded quartz dishes.

Cells were attached to the surface of quartz (control) dishes at day one, but started to shrink and aggregate from day 2. At day 4, most of the cells retracted and detached from the quartz dish surface and underwent apoptosis. In contrast, quartz dish surfaces coated with nanodiamond powders showed cells attached well to the plate at day 1 (Figure 3) and started to shrink and aggregate. The width of the images of 10× is 0.695 mm and the width of the images of 4× is 1.738 mm. At day 4, most of the cells were still attached to the surfaces of quartz dishes coated with nanodiamond powders.

**Figure 4:**
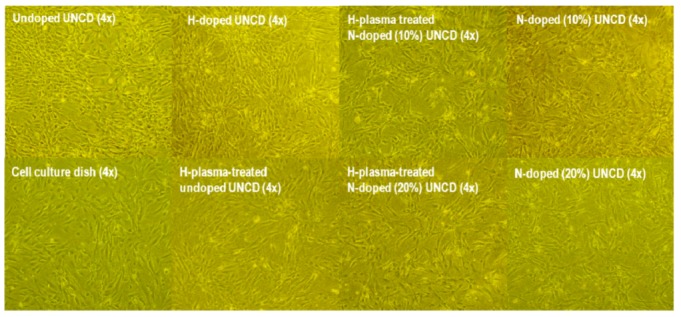
Comparison of MEF growths on different UNCD films at day 4.

Cell growth on undoped UNCD, H-plasma-treated UNCD, H-doped UNCD, N-doped UNCD (10%), H-plasma-treated N-doped UNCD (10%) thin films, N-doped UNCD (20%), and H-plasma-treated N-doped UNCD (20%) thin film coated dishes showed good cell attachment at the first day and started to proliferate normally. At day 4, cell growth reached confluency and cells were morphologically normal (Figure 4).

**Figure 5:**
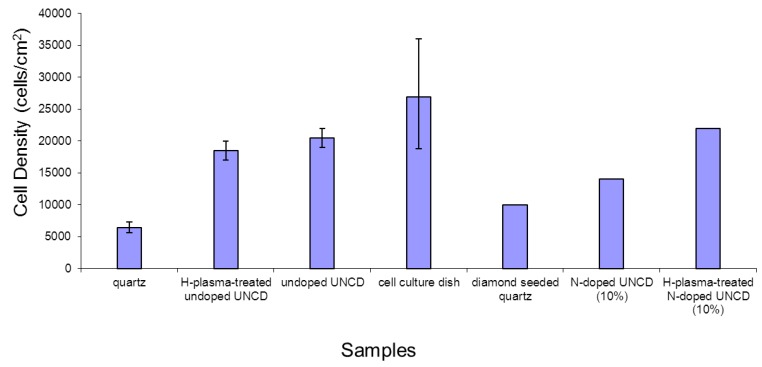
Comparison of cell densities at day 4.

Cells were dissociated at day 4 and the total numbers of cells from each growth condition were counted. The results are shown in Figure 5. Cell culture dishes had the highest densities, yet with the largest variation among three replicates of the cell culture dishes, quartz dishes, undoped UNCD dishes, and the H-plasma-treated undoped UNCD dishes. Quartz dishes had the lowest cell density. Diamond-seeded quartz dishes showed the second lowest densities. 10% and 20% N-doped UNCD thin film coated dishes had similar densities of cells. Different types of UNCD-coated dishes had different densities without significant differences.

### 3.4. Matrix-Assisted Laser Desorption/Ionization—Time-of-Flight Mass Spectrum (Maldi-Tof Ms)

**Figure 6:**
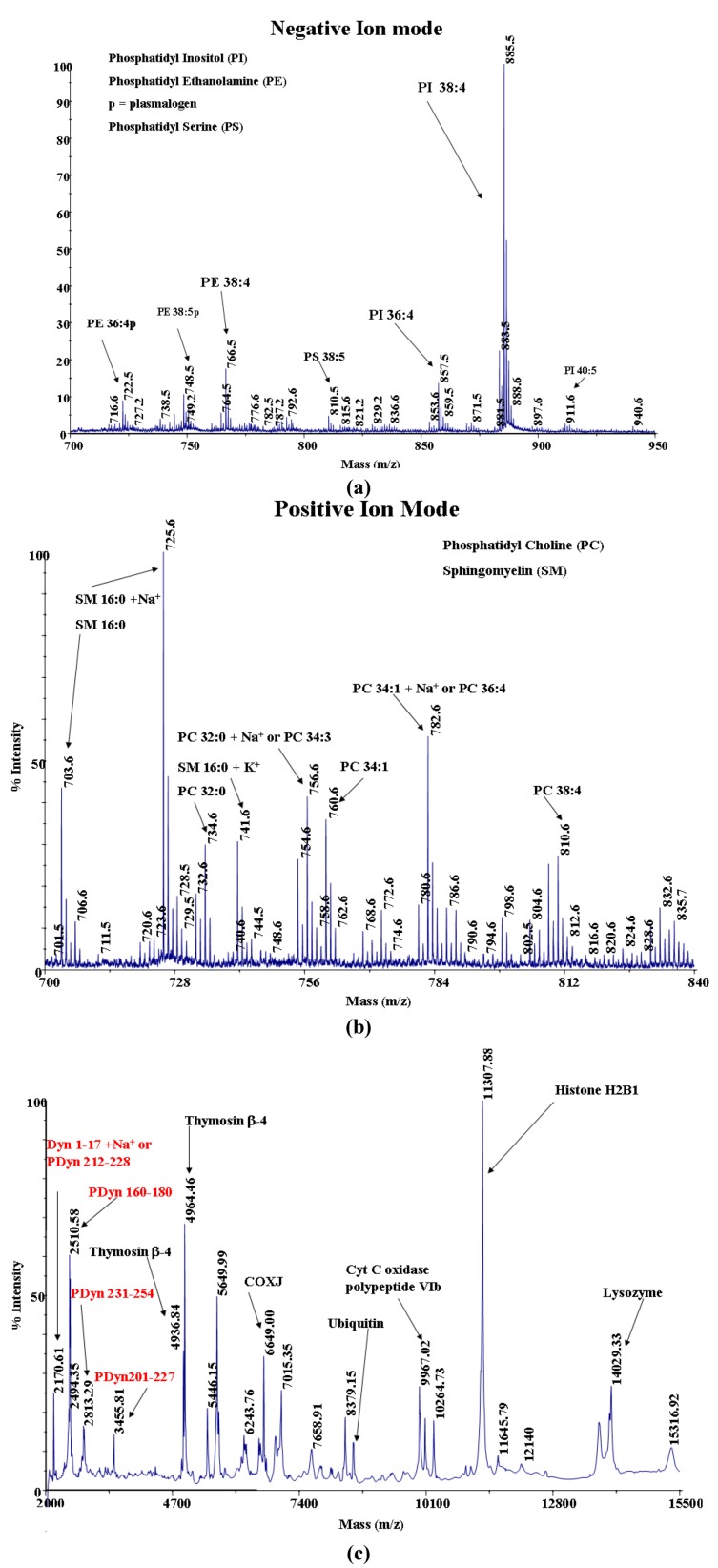
Matrix-assisted laser desorption/ionization (MALDI) analysis on MEFs. (**a**) Negative ion mode analysis; (**b**) Positive ion mode analysis (mass: 700–840 m/z); (**c**) Positive ion mode analysis (mass: 2,000–15,500 m/z).

**Figure 7:**
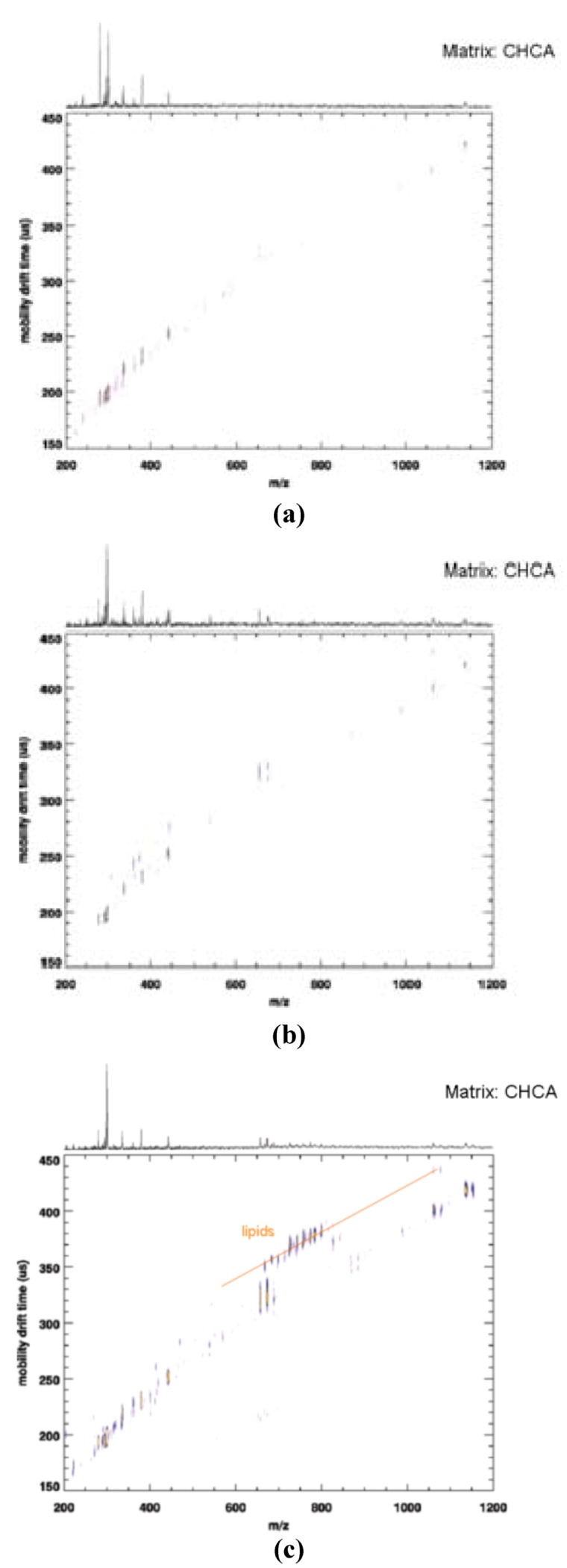
MALDI analysis of MEFs on UNCD. (**a**) UNCD; (**b**) UNCD in buffer; (**c**) MEFs on UNCD in buffer.

MALDI-TOF MS analyses were done on MEFs and MEFs on a UNCD surface. Results are shown in Figure 6 and Figure 7. Analysis of the MEFs in Figure 6 showed that among the four major phospholipids predominating in the plasma membrane of many mammalian cells, phosphatidyl ethanolamine (PE), phosphatidyl serine (PS), phosphatidyl choline (PC), and sphingomyelin (SM), PE and PS are negatively charged while PC and SM are positively charged. Among the negatively charged biomolecules on the MEF membrane, phosphatidyl inositol (PI) has the largest intensity compared with PE and PS. 

PI is in the outer lipid monolayer of the plasma membrane. PI exposed at the external cell surface attaches only by a covalent linkage via a specific oligosaccharide to phosphatidylinositol. It plays a crucial role in the cell signaling. Of the positively charged biomolecules, both PC and SM have a large intensity. There are many other biomolecules that are also positively charged. Figure 7 shows the results of MEFs on UNCD. While the spectra of the blank UNCD film and the UNCD film exposed to the buffer solution are fairly similar, the UNCD film with the MEFs shows a series of strong peaks that should be interpreted as lipid ion peaks. These lipids most likely belong to the cell membranes. No high-mass proteins were detected due to the low amount of these species exposed to desorption and ionization by MALDI.

### 3.5. X-Ray Photoelectron Spectroscopy (XPS)

**Figure 8:**
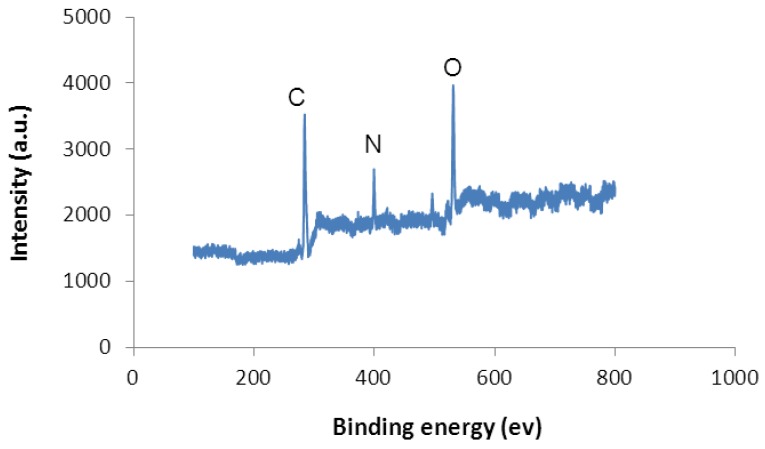
X-Ray Photoelectron Spectroscopy (XPS) analysis of cells on UCND.

Similar results from XPS analysis are shown in Figure 8. While UNCD showed only carbon peaks, both nitrogen peaks and oxygen peaks were detected after MEFs grew on UNCD. These peaks came from the membrane of the cells.

## 4. Discussion on Cell Attachment and Proliferation

Cells did attach to the quartz dishes at day 1, however, after day 1 cells started to detach and aggregate. If cells cannot deposit their extra-cellular matrix (ECM) on the substrate surface within a short period of time (24–48 hours), they undergo apoptosis [18]. The ECM that MEFs secreted did not deposit on the quartz dishes and therefore cells on quartz dishes started to undergo apoptosis. 

All of the different types of UNCD thin films investigated could support cell attachment and proliferation. UNCD thin films have a polycrystalline structure with grains of 2–5 nm characterized by sp^3^ carbon bonds, and grain boundaries about 0.4 nm wide characterized by a mixture of sp^2^ and sp^3^. The particular nanostructure of UNCD films results in a high density of grain boundaries, which may play a critical role in promoting cell growth, because the sp^2^ bonds along the nanocrystalline boundaries are active and make the UNCD surface electron-negatively charged. 

This hypothesis is further supported by the cell growth on diamond-seeded quartz dishes. Cells attached to the dishes at day 1. Small amounts of cells started to detach and aggregate, which led to the death of these cells. Other cells proliferated on the seeded quartz surface, which suggests the diamond seeds provided the environment that cells could live on. But the cells on seeded quartz dishes did not reach the same confluency as on the other UNCD-thin-films-coated dishes. Diamond-seeded quartz dishes could be described as the non-continuous UNCD grains. Cells that attached and proliferated on these seeded quartz dishes showed that the grain boundaries did provide the binding sites for cells. Cells that partially detached and aggregated on these seeded dishes did so because the seeds were not as continuous as the UNCD thin films, and therefore the cells were exposed to the quartz surface, which led to the death of these cells. 

**Figure 9:**
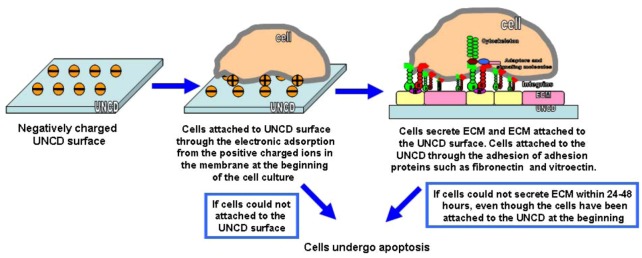
Schematic models of cell adhesion mechanisms to UNCD.

Besides the special microstructure and bonding information around the grain boundaries, the surface chemistry of UNCD films provided the binding sites for cells. Immediately after the synthesis of UNCD films finished, there was a substantial number of dangling bonds that were unsatisfied. Once the chamber was vented to the atmosphere, the dangling bonds on the surface of the films reacted with oxygen in the air and formed C-O bonds. In addition, the fresh synthesized UNCD surfaces absorbed water molecules in the air, leading to the formation of carboxy groups confirmed by the XPS analysis. The extra-cellular matrix contained a variety of versatile proteins and polysaccharides that could anchor on top of UNCD. MALDI analysis of the membrane of the MEFs and the analysis of MEFs on UNCD gave further evidence in support of the mechanisms described here. A schematic of the cells’ adhesion mechanism on UNCD is presented in Figure 9.

The surface chemistry of UNCD films plays a critical role, as demonstrated by the comparison of the cell growth on UNCD-film-coated dishes. Among the UNCD-film-coated dishes, the N-doped showed the lowest cell density. H-plasma treated N-doped UNCD-coated dished showed the largest cell density among the UNCD-coated dishes. As mentioned earlier, H-plasma treatment generated the H-terminated surface, which provided the most inertness on the undoped UNCD surface. On the N-doped UNCD, H-plasma treatment also generated H-terminated surface, and therefore, in addition to C-H bonds, other bonds such as N-H, and NH_2_ could also be formed on the surface, providing more binding sites for cell attachment and resulting in a higher cell density. Undoped UNCD films grown on quartz dishes showed the second largest density. The H-plasma treated undoped UNCD-coated dishes showed a lower density compared with the undoped UNCD surface.

## 5. Conclusions

Undoped, H-doped, and N-doped UNCD films were synthesized and H-plasma treatment was introduced to these films. MEF cell growth occurred on these different UNCD-coated dishes as well as on diamond-seeded quartz dishes. Results showed all these different UNCD-film-coated dishes could support cell adhesion and proliferation. The H-plasma treated N-doped UNCD-coated dishes showed the largest cell density among all UNCD-coated dishes because the surface introduced the NH_2_ group and because the unique microstructure could promote cell attachment and growth. The results were analyzed and the mechanisms of cell adhesion on the UNCD surfaces were proposed. The unique nanostructure of UNCD, with a high density of grain boundaries, plays a critical role in cell adhesion via chemical bonds such as hydrogen bonds, polar interactions, and electrostatic interactions between receptor molecules on the cell membrane and the chemical functional groups of the artificial substrates.
